# Liver Bacterial Dysbiosis With Non-Tuberculosis Mycobacteria Occurs in SIV-Infected Macaques and Persists During Antiretroviral Therapy

**DOI:** 10.3389/fimmu.2021.793842

**Published:** 2022-01-10

**Authors:** Bridget S. Fisher, Katherine A. Fancher, Andrew T. Gustin, Cole Fisher, Matthew P. Wood, Michael Gale, Benjamin J. Burwitz, Jeremy Smedley, Nichole R. Klatt, Nina Derby, Donald L. Sodora

**Affiliations:** ^1^ Seattle Children’s Research Institute, Center for Global Infectious Disease Research, Seattle, WA, United States; ^2^ Center for Innate Immunity and Immune Disease, Department of Immunology, University of Washington, Seattle, WA, United States; ^3^ Vaccine and Gene Therapy Institute, Oregon Health & Science University, Beaverton, OR, United States; ^4^ Oregon National Primate Research Center, Oregon Health & Science University, Beaverton, OR, United States; ^5^ Department of Surgery, University of Minnesota, Minneapolis, MN, United States

**Keywords:** HIV/SIV, microbiome, liver, 16S rRNA gene, neutrophils

## Abstract

Liver disease is a significant contributor to morbidity and mortality in HIV-infected individuals, even during successful viral suppression with combination antiretroviral therapy (cART). Similar to HIV infection, SIV infection of rhesus macaques is associated with gut microbiome dysbiosis and microbial translocation that can be detected systemically in the blood. As microbes leaving the intestines must first pass through the liver *via* the portal vein, we evaluated the livers of both SIV-infected (SIV+) and SIV-infected cART treated (SIV+cART) rhesus macaques for evidence of microbial changes compared to uninfected macaques. Dysbiosis was observed in both the SIV+ and SIV+cART macaques, encompassing changes in the relative abundance of several genera, including a reduction in the levels of *Lactobacillus* and *Staphylococcus*. Most strikingly, we found an increase in the relative abundance and absolute quantity of bacteria within the *Mycobacterium* genus in both SIV+ and SIV+cART macaques. Multi-gene sequencing identified a species of atypical mycobacteria similar to the opportunistic pathogen *M. smegmatis*. Phosphatidyl inositol lipoarabinomannan (PILAM) (a glycolipid cell wall component found in atypical mycobacteria) stimulation in primary human hepatocytes resulted in an upregulation of inflammatory transcriptional responses, including an increase in the chemokines associated with neutrophil recruitment (CXCL1, CXCL5, and CXCL6). These studies provide key insights into SIV associated changes in hepatic microbial composition and indicate a link between microbial components and immune cell recruitment in SIV+ and SIV+cART treated macaques.

## Introduction

HIV infection continues to be a major public health concern with approximately 37.6 million people living with HIV at the end of 2020 (1). With the introduction of highly effective combination antiretroviral therapy (cART), the life expectancy of HIV infected (HIV+) individuals has moved closer to that of the general population, particularly in higher income countries (2, 3). Nevertheless, HIV+ individuals experience a greater burden of co-morbidities, often at a markedly younger age, including cardiovascular disease, frailty, cognitive decline, and liver disease (4–6). Among these chronic health conditions, liver disease is especially prevalent, most notably non-alcoholic fatty liver disease (NAFLD), and is a leading cause of death in HIV+ individuals (7, 8). The factors that initiate hepatic lipid deposition, inflammation and the development of liver disease during HIV infection are poorly defined and are likely multifactorial. Several studies have linked HIV viral load to liver disease (9–11), suggesting that viral stimulation (either as a pathogen associated molecular pattern (PAMP) or through replication) plays a role. Liver macrophages (Kupffer cells) have been implicated in the inflammation associated with HIV infection due to alterations in the induction of inflammatory genes such as cytokines and chemokines (12). Liver disease can also be observed in HIV+ persons virally suppressed with cART (13), indicating that the virus itself is not the only driver of liver dysfunction. Although cART itself has previously been associated with hepatotoxicity (14, 15), NAFLD has continued to be an issue in HIV patients taking more recently developed and less hepatotoxic cART regimens (16). Importantly, liver disease in HIV+ individuals is complicated by the presence of comorbidities and coinfections which makes drawing of mechanistic conclusions more difficult and raises the importance of using an animal model to address these types of questions.

HIV+ individuals may be co-infected with a number of microbial invaders, including opportunists such as non-tuberculosis mycobacteria (NTMs). The gastrointestinal tract represents an important source of bacteria that can enter the circulation. After leaving the gastrointestinal tract, blood is filtered by the liver to remove key nutrients and bacteria prior to entering the general circulation. In healthy individuals, the bacterial products that enter the liver are associated with immune tolerance and low levels of inflammation (17). However, elevated bacterial load or altered bacterial composition (dysbiosis) can result in an inflammatory response within the liver (17, 18). Central to this inflammation are the Kupffer cells that play a key role in the clearance of microbial products from portal blood (19). Upon engagement of innate receptors [e.g. toll-like receptors (TLRs)] on these cells by microbial products, inflammatory and profibrotic mediators are produced, such as TNF-α, IL-12, IL-6 and TGF-β (19–21). Other cells involved in the inflammatory response are neutrophils, which are recruited and release reactive oxygen species and pro-inflammatory cytokines (18, 22), and hepatocytes that experience altered gene expression and production of inflammatory mediators (23, 24). Consistent with these findings, murine and clinical studies have established that microbial dysbiosis and leaky gut are both features of alcoholic liver disease and NAFLD, and increased levels of bacterial products have been identified in the livers and plasma of individuals with these conditions (17, 25–29). The association between bacterial translocation, systemic immune activation, and NAFLD with disease progression in HIV+ individuals (30) suggests that microbial dysbiosis may be a key driver of inflammation in HIV-associated liver disease.

The SIV-macaque model has played key roles in unraveling HIV associated disease outcomes, including establishing that liver inflammation and fibrosis occur in the absence of confounding triggers such as hepatitis virus infection (31). The SIV model has also established that bacterial translocation is associated with systemic immune activation observed during HIV disease progression (32). The gut microbiome of macaques is influenced by numerous factors including age, environment, food, antibiotics and SIV infection (33–38). Studies from our laboratory and others have identified increased levels of the bacterial products *E. coli*, LPS, and 16s rRNA DNA in the liver during SIV infection (39–41), and in the context of cART (32, 41). Dysbiosis of the rhesus macaque liver microbiome during SIV infection has previously been associated with an increase in Proteobacteria originating from the large intestine (32). Studies described here expand upon the earlier work by assessing liver microbial changes in both SIV-infected (SIV+) and SIV+ cART-treated (SIV+cART) macaques at lower taxonomic levels than evaluated previously, and by testing the impact of the prevalent observed microbial species on hepatocyte signaling. These studies, which use livers from macaques already characterized with regard to immune cell subsets by our laboratory (41), reveal novel patterns of liver microbial dysbiosis and immune responses that shape our understanding of HIV-associated liver disease.

## Materials and Methods

### Ethics Statement

All animal studies were directed in accordance with protocols approved by the Center for Infectious Disease Research (now Seattle Children’s Research Institute; CIDR protocol DS-05 UW), and Washington National Primate Research Center (WaNPRC), Seattle, WA (protocols 4314–01, 4213–02 and 4213–03) under the Institutional Animal Care and Use Committees (IACUCs). All rhesus macaques involved in this study were managed according to the laws, regulations, and guidelines set forth by the United States Department of Agriculture, Institute for Laboratory Animal Research, Public Health Service, National Research Council, Centers for Disease Control, the Weatherall Report titled “The use of nonhuman primates in research”, and the Association for Assessment and Accreditation of Laboratory Animal Care (AAALAC) International. Nutritional plans utilized by WaNPRC consisted of standard monkey chow supplemented with a variety of fruits, vegetables, and other edible objects as part of the environmental enrichment program established by the Behavioral Management Unit. Enrichment was distributed and overseen by veterinary staff with animals having access to more than one category of enrichment. SIV+ macaques were kept in individual, adjoining cages allowing for social interactions with primate health observed daily by trained staff. All efforts were made to minimize suffering using minimally invasive procedures, anesthetics, and analgesics when deemed appropriate by veterinary staff. Animals were painlessly euthanized by sedation with ketamine hydrochloride injection followed by intravenous barbiturate overdose following the recommendations of the panel of euthanasia of the American Veterinary Medical Association. These macaques have been described previously (41).

### Liver Tissue Collection

Liver tissue was collected at necropsy from uninfected (N=4), SIV+ (N=6) and SIV+ cART (N=6) adult Indian rhesus macaques (*Macaca mulatta*). Control samples from uninfected macaques were acquired from the Tissue Donor Program at WaNPRC. SIV+ macaques were infected intrarectally with SIVmac239x (41). SIV+ macaques receiving cART were administered subcutaneous tenofovir (20 mg/kg body weight) and emtricitabine (30 mg/kg) and oral raltegravir (50 mg twice daily) starting 120 days post-infection and continuing for 35-36 weeks prior to euthanasia (41). Care was taken during necropsies to prevent cross-contamination between samples and between macaques. Tissue was washed in phosphate buffered saline (PBS), formalin-fixed, paraffin-embedded for microscopy or flash-frozen in liquid nitrogen and then stored at -80°C for nucleic acid extraction.

### Liver Tissue Disruption by Ball Mill Pulverization

Flash-frozen liver tissue was pulverized into a fine powder by ball milling with three large stainless steel balls per sample in the stainless steel chamber of the Retsch Planetary Ball Mill under cryogenic conditions with liquid nitrogen (Retsch Laboratory Equipment, Haan, Germany). Each sample was subjected to three cycles at 300 rpm for two minutes each. Between each cycle, the chamber was removed from the ball mill instrument and re-frozen in liquid nitrogen to preserve cryogenic conditions. Following pulverization, the pale pink liver powder was collected using a spatula to retrieve the powder off the walls of the chamber and the balls into a collection tube (spatula and tube were both pre-chilled in liquid nitrogen) and stored at -80°C until DNA extraction. Machine and steel balls were cleaned between samples to prevent cross contamination.

### Liver Tissue Disruption and Phase Separation Using Bead Beater

DNA extraction was also undertaken following use of a bead beater MagNA Lyser bead beater (Roche Life Science, Penzberg, Germany). Frozen liver tissue was placed in a lysing tube containing ceramic beads (Lysing Matrix D, MP Biomedicals, Santa Ana, CA) with guanidine thiocyanate and phenol (Tri Reagent, Molecular Research Center, Cincinnati, OH) and dissociated at 6500 rpm for 45 seconds. The supernatant was incubated with 1/10 volume of bromochloropropane (Molecular Research Center) for 5 minutes and the organic and aqueous phases separated by centrifugation at 12,000xg for 15 minutes at 4°C.

### Tissue DNA Extraction From Liver Powder

Liver powder (10-30 mg) was placed into a sterile, pre-chilled microcentrifuge tube. Genomic DNA was extracted using the NucleoSpin Tissue DNA extraction kit (Takara, Mountain View, CA) per the manufacturer’s instructions, where samples were pre-lysed and allowed to incubate at 56°C for at least 1-3 hours, mixing occasionally. Samples were then lysed with provided buffer, vortexed vigorously, and incubated at 70°C for 10 minutes. Ethanol was added and samples were centrifuged in NucleoSpin Tissue Columns into a collection tube at 11,000 x g for 1 minute. After a series of washes, samples were eluted with elution buffer and collected. Following concentration determination with a NanoDrop 2000 Spectrophotometer (Thermo Scientific, Waltham, MA), isolated genomic DNA was stored at -80°C until use.

### 16s rRNA Gene Sequencing and Microbiome Analysis

Genomic DNA extracted from the liver (20 µL) was used for 16s rRNA gene community sequencing through Illumina according to the Earth Microbiome Protocol (42). The V3-V4 region of the 16s rRNA gene was amplified in triplicate using 515fB and 806rB primers at 0.2uM. Cycling conditions were 94°C for 3 minutes followed by 35 cycles of 94°C for 45 seconds, 50°C for 60 seconds and 72°C for 90 seconds, followed by a final extension at 72°C for 10 minutes. PCR amplicons were cleaned with 0.8x AMPure XP beads (Beckman Coulter, Brea, CA) before the addition of Nextera XT dual index adaptors (Illumina Inc., San Diego, CA). Indexed amplicons were cleaned using 1.1× AMPure XP beads (Beckman Coulter, Brea, CA), quantified using a Qubit DNA high-sensitivity assay kit (Life Technologies, Carlsbad, CA), and multiplexed using an equal molar ratio of DNA for each sample. 16S rRNA gene libraries were loaded on a 300-cycle MiSeq kit and sequenced using Nextera sequencing read and index primers (all from Illumina Inc.). Paired-end demultiplexed FASTQ files from the Illumina base space were imported into the QIIME2 pipeline (QIIME 2 Core 2019.10) to create a demultiplexed QIIME2 object. These objects were matched to identified amplicon sequence variants (ASVs) using the dada2 algorithm which worked to detect and correct Illumina amplicon sequence data and denoise by trimming to 145 bases to remove low-quality regions. A rooted phylogenetic tree was constructed using the Mafft multiple sequencing alignment program and taxonomy was assigned using the SILVA database specific to the V3-V4 region. After taxonomy was determined, results were exported from the pipeline for downstream analysis in R using the phyloseq package.

### Quantification of Mycobacterial DNA in the Liver by qPCR

All liver DNA samples were diluted in nuclease-free water. Each sample (5 µL) was prepared in duplicate in a 20 µL volume reaction with the PowerUp SYBR Green Master Mix kit (Applied Biosystems, Waltham, MA) and *Mycobacterium*-specific primers (MycoARB210: 5’-TTT GCG GTG TGG GAT GGGC-3’ and MycoARB585: 5’-CGA ACA ACG CGA CAA ACCA-3’). A ‘No Template’ Negative Control was included to control for reagent contamination and non-specific amplification. A standard curve was generated by serially diluting pure *M. bovis* (BCG) DNA 10-fold, ranging from 10-0.001 ng/µL (R^2^ > 0.95). PCR reactions ran one cycle at 50°C for 2 minutes then increasing to 95°C for 2 minutes followed by 45 cycles of 94°C for 15 seconds, annealing at 61°C for 30 seconds, and extending at 72°C for 30 seconds with a final extension step at 72°C for 7 minutes. Following the qPCR cycles, PCR reactions were subjected to a melt curve analysis to examine products formed. The concentration of *Mycobacterium* DNA per sample was determined through a non-linear regression on the standard curve and converted to copy number based on BCG molecular weight (5.63x10^12^ mg/mole). The weight was then converted to 4.277x10^7^ molecules/mole and the standard curve was plotted based on molecules where copy number was equal to 4.277x10^7^ molecules/mole * log (CT) where the standard curve equation was extrapolated (y = -0.032ln(x) +1.8377). Copy number of the liver *Mycobacterium* DNA was then calculated from the standard curve equation (copy number = e ((log (Ct) – 1.8377)/-0.032)). Duplicates were averaged for each sample. qPCR to detect a conserved region of the *16S rRNA* gene was performed as reported previously (41).

### Identification of *Mycobacterium* Species by Multi-Gene Sequencing

Genomic DNA (gDNA) was diluted in nuclease-free water and amplified by nested PCR in the *16s rRNA* and the *rpoB* genes per the conditions outlined in **Supplementary Table 1**. For each first-round PCR reaction, 500 ng (5 µL) of gDNA was added into a 50 µL reaction and amplified using the Platinum *Taq* DNA Polymerase reaction kit (Invitrogen, Carlsbad, CA). For nested PCR reactions, 1 µL of the first-round PCR product was added to a 50 µL reaction containing the nesting primers and Platinum *Taq* DNA Polymerase. Each round of PCR contained a positive control of BCG DNA and a negative no template (water) control. Amplicons from the nested PCR were examined by 1% agarose gel. Each PCR amplicon showing the correct size was cleaned up using a Nucleospin PCR Clean-up Kit (Takara) and eluted into 30 µL of EB buffer. Purified PCR amplicons (20 ng) along with positive and negative control samples were sequenced by Sanger sequencing using both forward and reverse nesting primers in separate reactions. Following sequencing, DNA sequence quality was examined in 4Peaks software and low-quality reads from the 5’ and 3’ ends removed. Consensus sequences generated through the alignment of forward and reverse reads were analyzed with BLAST.

### Culture and Stimulation of Human Hepatocytes With Mycobacteria and Mycobacterial Antigens

Human HepaCure Hepatocytes on Matrigel overlay (350,000 hepatocytes/well in 24-well dishes) were acquired from Yecuris (Tualatin, OR). HepaCure human hepatocytes are produced by the immunization of humanized FRG®KO mice with cadaver-derived human hepatocytes. Upon receipt, the media was immediately replenished with 500 μL InVitro GRO Hi Medium (BioIVT, Westbury, NY) supplemented with Torpedo Antibiotic Mix (BioIVT). Cultures were incubated at 37°C, 5% CO_2_ overnight. To determine the hepatocyte response to mycobacterial PAMPs, purified lipoarabinomannan (LAM) from *M. smegmatis* (PILAM, 0.1 and 10 ug/mL, BEI Resources, Manassas, VA), or *M. tuberculosis*, Strain H37Rv (ManLAM, 0.1 and 10 ug/mL, BEI Resources), were added to hepatocytes. Each stimulation condition, as well as the control condition, were conducted in triplicate. Plates were incubated at 37°C, 5% CO_2_ for 24 hours. For live mycobacteria stimulations, *M. smegmatis* bacteria (strain MC^2^155) were grown to exponential phase, washed with PBS and resuspended in InvitroGRO Hi Medium without antibiotics at 350,000 bacteria/μL. Hepatocytes were stimulated with *M. smegmatis* (multiplicity of infection (MOI) 10) in triplicate for 24 hours at 37°C, 5% CO_2_. For all stimulations, supernatant was collected and stored at -80°C. The hepatocyte monolayer was then washed with 500 μL pre-warmed PBS and then lysed in 300 μL RA1 buffer containing beta-mercaptoethanol. RNA was isolated from the cell lysate following protocols from the NucleoSpin RNA isolation kit (Macherey-Nagel, Bethlehem, PA).

### Transcriptomic Analysis of HepaCure Hepatocytes by Nanostring

RNA (triplicates from the stimulation experiment) was diluted to 20 ng/μL in nuclease-free water and used for transcriptomic analysis using a Nanostring Inflammation Panel (Human v2) (Nanostring, Seattle, WA). Probe set-target RNA hybridization reactions were performed according to the manufacturer’s protocol using 100 ng (5 μL) of total RNA. Purified probe set-targets were processed and immobilized on nCounter Cartridges using a nCounter MAX prep station. Transcripts of interest were quantified on the Digital Analyzer for each sample. For data analysis, nCounter RCC files were imported in nSolver Analysis Software 4.0 and checked for quality control. Determination of differentially expressed genes, pathways analysis, and cell profiling was conducted using the Nanostring Advanced Analysis software per the manufacturer’s instructions. For each stimulation condition, differentially expressed genes were determined by comparing the normalized count data between stimulated hepatocytes and unstimulated control hepatocytes. Heatmaps were generated in Prism version 5.0f software (GraphPad Software, Inc., San Diego, CA), showing fold change of each gene in the panel. Volcano plots were assessed using the Python Matplotlib package for significant genes using a threshold of 1.5-fold change (log2(1.5) = 0.585) and 0.05 adjusted p-value.

### Statistics

Statistical analyses were performed using Prism version 5.0f software (GraphPad Software, Inc.). A nonparametric ANOVA (Kruskal Wallis) test was used to compare the uninfected, SIV+, and SIV+cART groups with post-test pairwise comparisons conducted using the Dunns test. Transcriptomic analysis was completed using nSolver (Nanostring, version 4.0.62).

## Results

### The Liver Microbiome During SIV Infection

**Figure 1:**
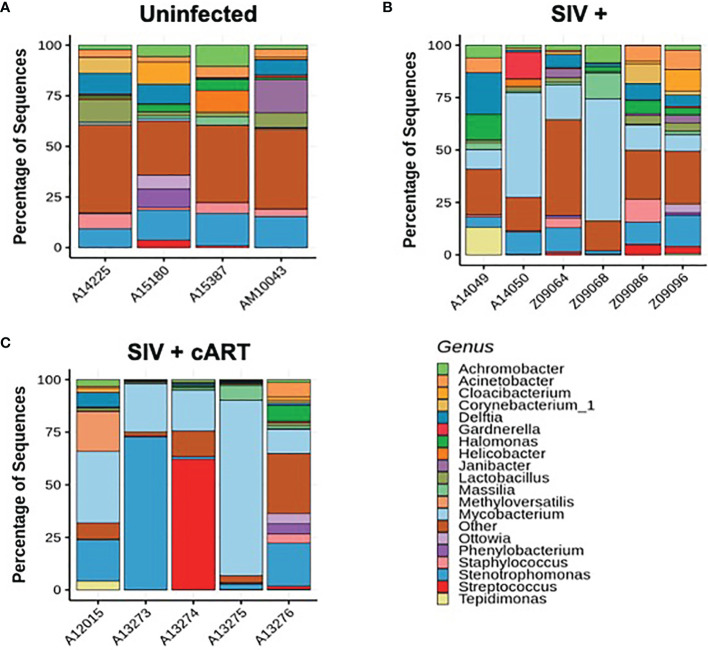
Individual macaque liver 16S microbiome analysis. Amplified 16S bacterial DNA sequences from liver samples were analyzed using the QIIME2 pipeline and visualized as percentage of sequences present in individual samples for **(A)** Uninfected macaques, **(B)** SIV+ macaques, and **(C)** SIV+cART macaques. Only bacteria representing the nineteen most abundant genera are included; other genera are compiled into the “Other” category.

The liver microbiome was evaluated at the genus level in macaques that were uninfected, SIV+, and SIV+cART through 16S rRNA gene sequencing (41). Liver bacterial communities displayed variation overall among the macaques, and the observed taxa represented several phyla: Actinobacteria, Bacteroidetes, Chlamydiae, Deinococcus-Thermus, Epsilonbacteraeota, Firmicutes, Fusobacteria, Proteobacteria, and Tenericutes (**Supplementary Figure 1**). Of the 49 genera identified across all the animals’ liver samples, almost half (22 of 49) represented Proteobacteria. Proteobacteria also made up 6 of the 10 genera with sequences present at greater than 10% prevalence in the complete cohort, in agreement with previous studies showing a high prevalence of Proteobacteria in the macaque liver (32). The remaining high prevalence sequences (>10% abundance) came from Firmicutes and Actinobacteria with Bacteroidetes also being well-represented (**Supplementary Figure 1**). Within these phyla, many genera were common across the liver microbial communities of the complete macaque cohort while others were present sporadically (**Figures 1A–C**).

**Figure 2:**
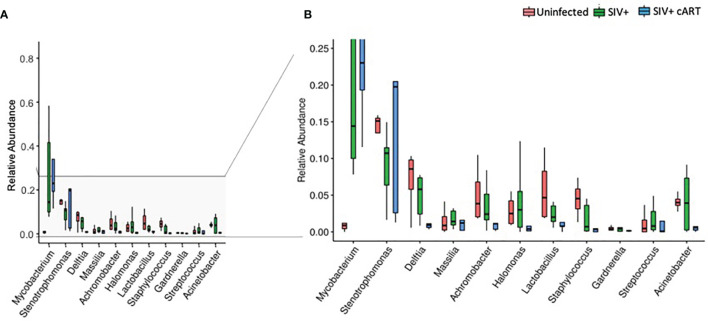
Relative abundance of identified genera in macaque groups. Relative abundance of the most prevalent genera across all liver samples identified by 16S rRNA gene sequencing compared by condition group: Uninfected, SIV+, and SIV+cART. Plots were generated using the pyloseq package by plotting mean fraction of sequences ± SEM for each condition group per genus and are depicted on a single scale **(A)** and with an expanded scale **(B)** that highlights the differences between less prevalent genera.

Uninfected macaques had a diverse liver microbiome with no dominant pervasive genus (**Figure 1A**). Within the uninfected macaques’ 19 most abundant genera, the highest percentage of sequences belonged to *Stenotrophomonas* (Proteobacteria phylum), and this genus constituted approximately 15% of sequences. In contrast, liver bacterial communities in the macaques from both the SIV+ and SIV+cART groups consisted of a greater abundance of sequences in the *Mycobacterium* genus (**Figures 1B, C**) [Actinobacteria phylum, median ~14% in SIV+ and ~20% in SIV+cART (**Figure 2**)]. Animals A14050 (SIV+), Z09068 (SIV+) and A13275 (SIV+cART) carried the highest percentage of *Mycobacterium* sequences (**Figures 1B, C**). Other SIV-related differences include decreased relative abundance of *Lactobacillus* and *Staphylococcus* (both Firmicutes phylum) in SIV+ macaques that persisted with cART as well as decreased relative abundance of *Halomonas* and *Acinetobacter* (both Proteobacteria phylum) that was specific to cART-treated SIV (**Figures 1**, **2**). Comparison of the median relative abundance across treatment groups revealed that the SIV+cART group exhibited an overall reduction in microbial diversity compared with the SIV+ group; all genera were reduced except for *Mycobacterium* and *Stenotrophomonas*, which were increased compared with the SIV+ macaques (**Figure 2**).

**Figure 3:**
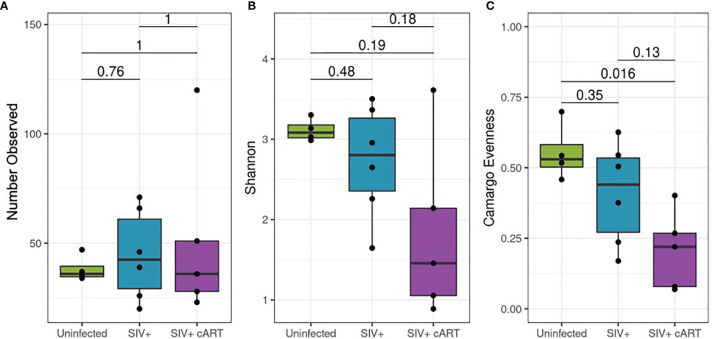
Alpha diversity of the 16S rRNA gene detected in liver. Amplified 16S rRNA gene sequences from liver samples were analyzed using the QIIME2 pipeline for alpha diversity and compared by condition group: Uninfected, SIV+ and SIV+cART. **(A)** Evaluation of richness of observed taxa across condition groups showing the mean (± standard deviation, SD) number of observed taxa within the condition group. **(B)** Representation of the proportion of species abundance showing the mean Shannon index (± SD) in the given population, where higher index indicates similar number of individuals. **(C)** Comparison of evenness among the condition groups, showing the mean (± SD) representation by different taxa.

Differences in alpha diversity metrics between the macaque groups followed from the observed differences in relative abundance (**Figures 3A–C**). The richness of observed microbial communities observed within each macaque liver (reflected as the number of observed taxa) were not significantly changed amongst the treatment groups (**Figure 3A**) while the most significant finding was the degree to which a single taxon dominated the liver microbiomes of SIV+cART macaques, which was significantly less even when compared to uninfected macaques (**Figure 3C**). Due to the disparity in evenness, the microbiomes of SIV+cART macaques also demonstrated the lowest Shannon diversity (**Figure 3B**), though this finding was not significant. Paralleling the variation observed specifically in *Mycobacterium*, SIV+ macaques had high variation in the overall number of observed taxa compared to the uninfected and SIV+cART groups (**Figure 3A**).

### Assessment of Mycobacterial DNA

**Figure 4:**
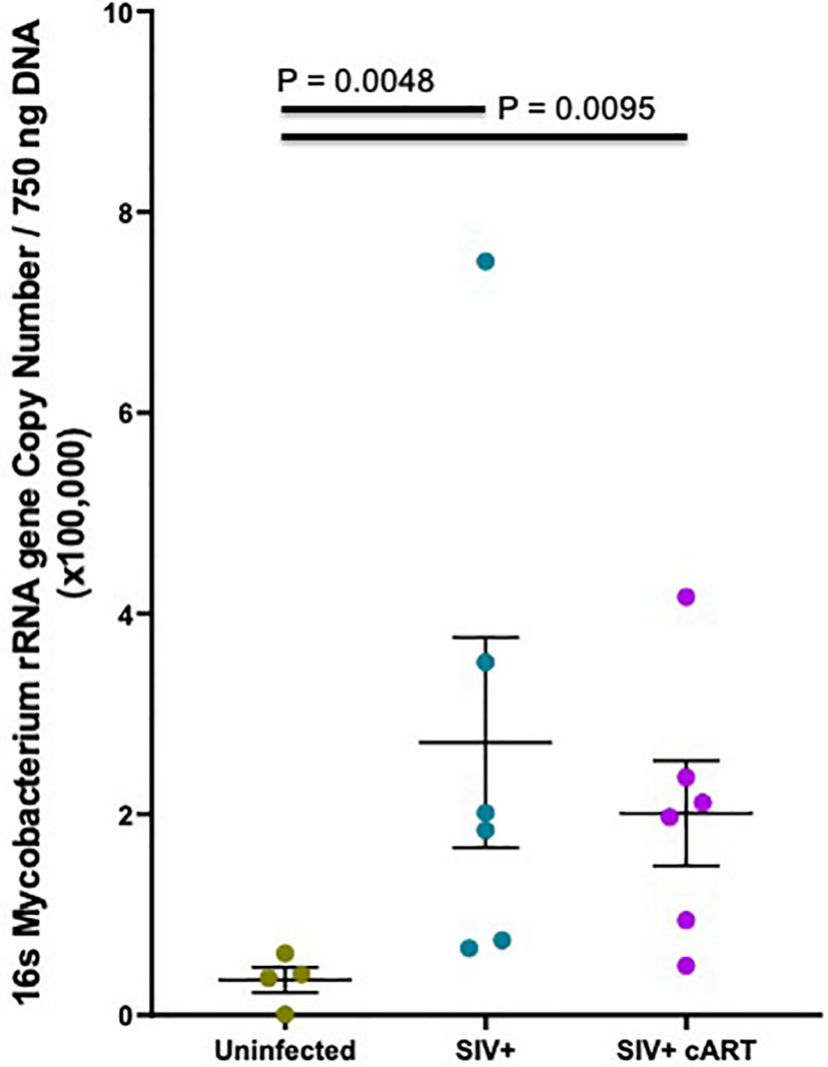
Hepatic *Mycobacterium* DNA quantitation by qPCR. Quantitative PCR was conducted with *Mycobacterium* specific 16S rRNA gene primers to quantify amounts of DNA present in macaque liver samples. *Mycobacterium* 16S rRNA DNA was quantified from a standard curve created by serially diluting pure *M. bovis* (BCG) DNA 10-fold and then converted into copy number per 750 ng template DNA based off of the molecular weight of BCG. Duplicates from each macaque were averaged and the mean plotted by treatment group with Uninfected macaques shown in gray, SIV+ macaques in teal, and SIV+cART macaques in purple. Statistical significance was determined using a Kruskal Wallis test and Dunns post-test with a significance cutoff of p<0.05.

Since 16S rRNA gene sequence abundance is a relative estimate that reflects the abundance of other bacteria, qPCR was conducted to confirm that *Mycobacterium* was indeed increased in the liver during SIV infection in both untreated and cART-suppressed macaques. Based on previous methods (43), extracted liver DNA was quantified in the liver of each macaque using *Mycobacterium 16S rRNA* gene-specific primers. *Mycobacterium* DNA was detected in all macaques, although levels in some of the uninfected macaques were extremely low. The copy number was significantly higher in both the SIV+ (p=0.0048) and the SIV+cART macaques (p=0.0095) when compared to uninfected macaques (**Figure 4**). These data confirm that *Mycobacterium* DNA was present in the liver during SIV infection as seen in the 16S rRNA gene sequencing, and that cART did not restore the liver microbiome to normal composition even after more than 30 weeks of viral suppression.

To better understand which *Mycobacterium* species were present in the livers of these macaques during SIV infection, multi-gene amplicon sequencing was performed. High sequence homology in closely related mycobacteria necessitates the use of multiple genes to help identify the species. Thus, *Mycobacterium*-specific primers for both the *16S rRNA* gene and the *rpoB* gene were utilized to amplify variable regions of each gene, followed by sequencing. Identification of liver mycobacteria using the *16S rRNA* gene indicated the presence of NTM of a few possible species, including *M. smegmatis, M. marinum*, or *M. goodii*, with greater than 99% sequence match (**Table 1**). Two of the macaques (Z09086, Z09096) yielded top BLAST hits exclusively for *M. smegmatis*. The *rpoB* gene has less sequence coverage in the BLAST database but is valuable in combination with the *16S rRNA* gene analysis. The *rpoB* gene sequencing analysis consistently yielded the identification of *M. smegmatis* in the liver of each macaque with >99% identity matches for all liver DNA samples tested, with exception to Z09096 which did not have enough DNA template for this secondary PCR verification (**Table 1**). Taken together, these data suggest that *M. smegmatis* or a closely related species is likely the specific *Mycobacterium* species present in the livers of these SIV+ and SIV+cART macaques.

**Table 1:** Identity of Liver *Mycobacterium* Species.

SIV+	16S rRNA Gene (% identity)	rpoB Gene (% identity)
A14049	*M. smegmatis, M. marinum* (99%)	*M. smegmatis* (99%)
A14050	*M. smegmatis, M. marinum* (100%)	*M. smegmatis* (99%)
Z09064	*M. smegmatis, M. marinum, M. goodii* (99%)	*M. smegmatis* (99%)
Z09068	*M. smegmatis, M. marinum* (99%)	*M. smegmatis* (100%)
Z09086	*M. smegmatis* (100%)	*M. smegmatis* (99%)
Z09096	*M. smegmatis* (99%)	QNS
**SIV+ cART**	**16S rRNA Gene (% identity)**	**rpoB Gene (% identity)**
A12015	*M. smegmatis, M. goodii* (99%)	*M. smegmatis* (99%)
A13273	*M. smegmatis, M. goodii, M. marinum* (100%)	*M. smegmatis* (99%)
A13274	*M. smegmatis, M. marinum* (99%)	*M. smegmatis* (99%)
A13275	*M. smegmatis, M. goodii, M. marinum* (100%)	*M. smegmatis* (99%)

QNS = DNA Quantity Not Sufficient for PCR verification

### Hepatocyte Transcriptional Responses to Mycobacteria Cell Wall Components

Aberrant hepatocyte inflammation and metabolism are key drivers in fatty liver disease. To explore the relationship between *Mycobacterium* and hepatocyte inflammatory responses, we stimulated primary human hepatocytes *in vitro* with purified LAM and assessed gene expression using a Nanostring inflammation panel (44). LAM is a major component of the *Mycobacterium* cell wall and a PAMP. Different species of *Mycobacterium* express distinct LAMs. Mannosylated LAM (ManLAM) is typically found in more pathogenic *Mycobacterium* species, such as *M. tuberculosis* while phosphoinositol-capped LAM (PILAM) is found in opportunistic *Mycobacterium*, such as *M. smegmatis*.

**Figure 5:**
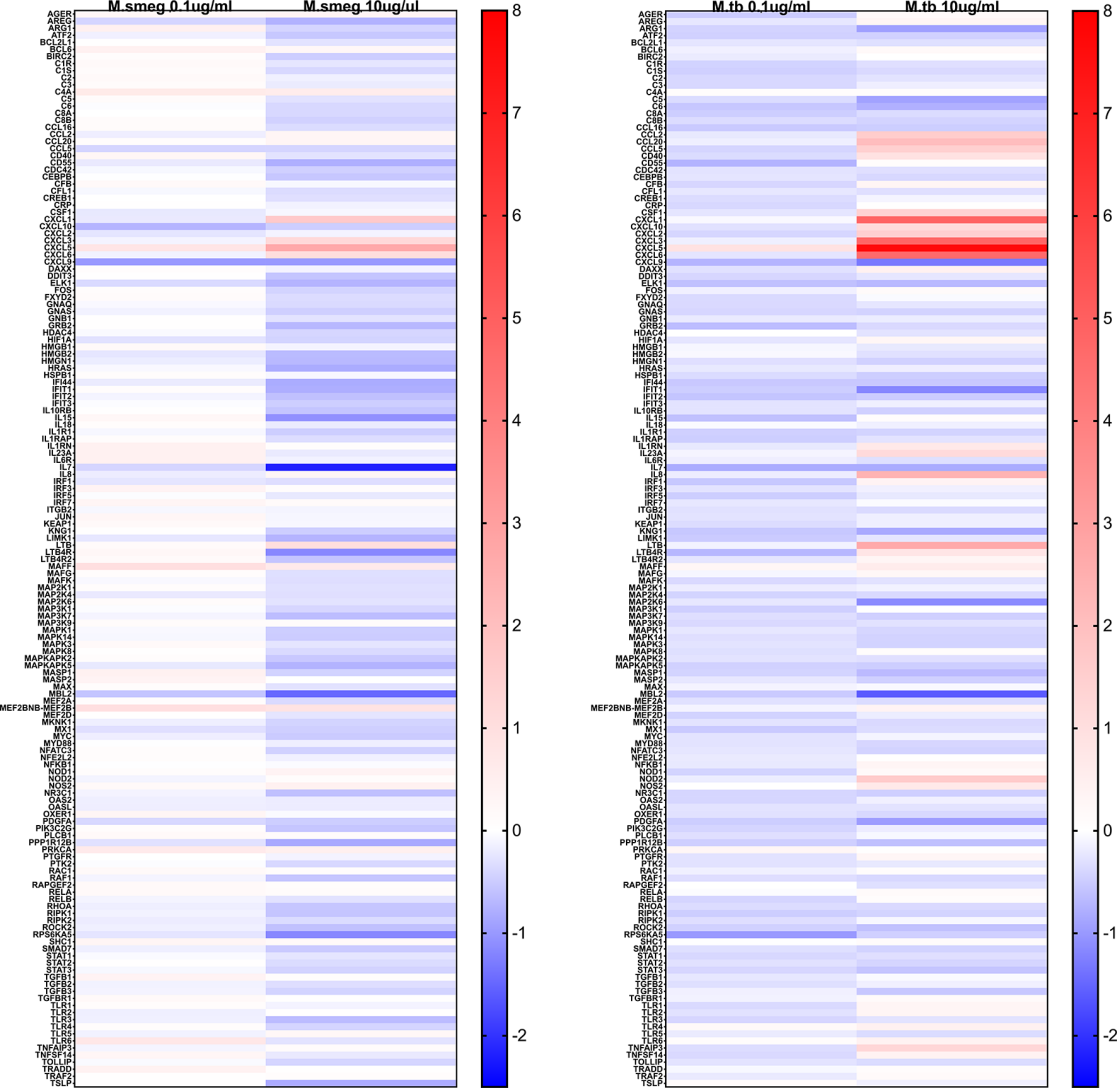
*Mycobacterium* LAM hepatocyte stimulation global inflammatory gene expression heatmap. Human HepaCure hepatocytes were stimulated with *M. smegmatis* PILAM (Left) and *M. tuberculosis* ManLAM (Right) at 0.1 mu/mL and 10 ug/mL for 24 hours. Extracted RNA was evaluated using the Human v2 Nanostring Inflammation Panel and compared using log2 fold change relative to the unstimulated control hepatocytes. Genes in red indicate an up-regulation and in blue a down-regulation. All genes included in the panel are represented on the heatmap.

**Figure 6:**
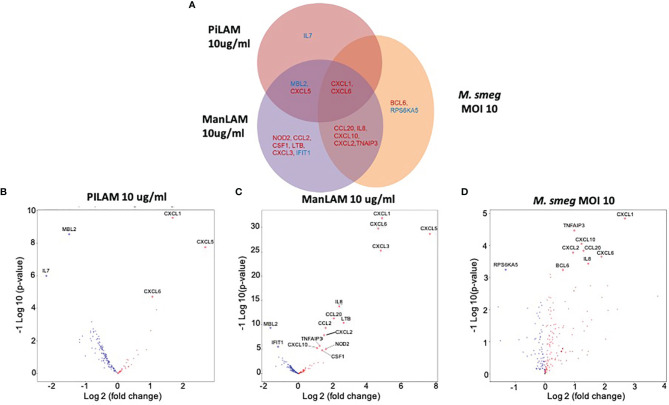
Comparison of *in vitro* hepatocyte transcriptomic response to mycobacterial PAMPs. Human HepaCure hepatocytes (Yecuris) were stimulated for 24 hours with live *M. smegmatis* (MOI 10), purified mycobacteria PILAM (10 mu/mL) obtained from *M. Smegmatis*, or purified mycobacteria ManLam (10 mu/mL) obtained from *M. tuberculosis*. Expression of inflammatory genes was quantified *via* a Nanostring gene expression panel. **(A)** Genes with a significant log2 fold change compared to the unstimulated control (p< 0.05, 1.5-fold change cut off) for each of the three stimulation conditions are displayed. Genes with significant expression changes common between stimulation conditions are placed in overlapping areas with up-regulated genes depicted in red and down-regulated genes in blue. **(B)** Volcano plot for gene expression in hepatocytes stimulated with PILAM (10 mu/mL) for 24 hours with significant genes denoted with labels and diamond points. **(C)** Volcano plot for gene expression in hepatocytes stimulated with ManLAM (10 mu/mL) for 24 hours with significant genes denoted with labels and diamond points. **(D)** Volcano plot for gene expression in hepatocytes stimulated with live *M. smegmatis* (MOI 10) for 24 hours with significant genes denoted with labels and diamond points.

Stimulation with LAMs induced a dose dependent global change in hepatocyte gene expression compared to the control condition (**Figure 5**). ManLAM and PILAM both altered expression of the same genes; however, overall ManLAM impacted gene expression more strongly than PILAM (**Figure 5**). An exception was *IL-7*, which was more dramatically down-regulated when hepatocytes were stimulated with PILAM (**Figures 6A, B**). Significant gene expression changes common between the ManLAM and PILAM stimulation include several chemokines involved in neutrophil chemotaxis (*CXCL1, CXCL6, CXCL5*) (**Figures 6A, B**), albeit with a fold change that was higher for hepatocytes stimulated with ManLAM (**Figure 6C**) compared to PILAM (**Figure 6B**). Comparison of the LAM stimulation with stimulation by live bacteria revealed that the hepatocyte transcriptome observed under stimulation with live *M. smegmatis* (MOI 10) more closely resembled that of hepatocytes stimulated with ManLAM (*M. tuberculosis*) than PILAM (*M. smegmatis*) (**Figure 6D**) including an upregulation in expression of *CCL20, CXCL10, CXCL2* and *IL-8*.

## Discussion

Different bacterial taxa have the potential to be inflammatory or anti-inflammatory; thus the composition of microbial communities within the body exerts important influences on homeostasis (17). Like HIV infection (45), SIV infection is associated with gut and liver microbiome dysbiosis, including an enrichment for inflammatory Proteobacteria that preferentially translocate out of the gut lumen into the colon and are detected within the liver (32). Microbial products that leave the gut *via* the portal circulation are subject to immune responses in the liver, such as inflammation and tissue damage in the case of increased bacterial load or dysbiotic taxa (39). In previous work, we identified an increase in the total amount of bacterial DNA in the livers of SIV+ macaques that persisted in the presence of effective cART and determined that the heightened level of bacterial DNA did not correlate with macrophage numbers, which instead correlated with plasma viral load and thus declined in cART-treated animals. Thus, here we sought to identify the taxa of translocated bacteria present in the liver and mechanistically assess the relationship between these bacteria and hepatic immune activation. Using bacterial 16S rRNA gene sequencing together with qPCR, we characterized the liver microbiota to the genus level and confirmed an increase in the most prolific genus, *Mycobacterium*, in SIV+ and SIV+cART macaques. Multigene PCR sequencing identified the *Mycobacterium* as *M. smegmatis* or a closely related species. Evaluating the impact of *M. smegmatis* on hepatocyte inflammation *in vitro* revealed that *M. smegmatis* and its cell wall component PILAM induce an upregulation of a number of cytokines and chemokines, including CXCL1, CXCL5, CXCL6, which are key for neutrophil recruitment.

The livers of SIV+ animals presented with an increase in prevalent genera during infection, with *Mycobacterium* as the most abundant genus present and persistent in the face of suppressive cART. In HIV+ patients, there is reduced diversity in gut microbiome composition that does not generally recover to pre-HIV levels after cART is initiated (45). Liver microbial communities of SIV+ macaques herein varied widely in their richness, suggesting that microbial effects of SIV infection may be host and context dependent and may influence the inter- and intra-genus diversity of the liver microbiome. SIV+ animals treated with cART possessed the least even spread of observed taxa with the opportunistic mycobacteria persisting and/or expanding. There are at least two plausible explanations for why cART did not substantially reduce the levels of atypical mycobacteria in the livers of SIV+ macaques. Livers were analyzed 35 to 36 weeks after the onset of cART, and this may be insufficient time for microbiome restoration. Alternatively, or in addition, clearance of mycobacteria may be difficult once colonization is established. Yet another possibility is that host conditions that promote dysbiosis such as metabolism are not restored during cART, thereby furthering the dysbiotic state. Longitudinal characterization of the liver microbiome during infection and at later time points following the introduction of cART as well as an understanding of the role of cART alone will aid in our understanding of these observations. Notably, the low detection of *Mycobacterium* in the uninfected macaques indicates that bacteria of this genus are likely present in the normal liver microbiome, but the immunocompromised state of SIV infection allows the bacteria to opportunistically thrive. Thus, SIV infection may provide the opportunity for specific genera that are present in the liver microbiome to increase in prevalence, rather than allow for the introduction of new genera. It is also notable that most of the genera identified in all macaque livers in this study were from the Proteobacteria phylum with Bacteroidetes and Firmicutes also highly represented in keeping with previous results in SIV+ macaques (32) as well as in obese humans (46), and thus it is likely that certain bacteria are simply more capable of translocation than others and are thus more likely to be detected in the liver.

Detection of mycobacteria in tissue samples is impeded by the hard-to-lyse cell wall of these microbes. Ball milling under cryogenic conditions provides high quality microbial DNA and efficiently retrieves DNA from mycobacteria. There are two reasons why we are confident that detection of mycobacteria DNA in the macaque liver samples processed by ball milling in this study represents a rigorously tested and well-controlled finding. First, analysis of liver samples using the ball mill revealed large differences between adjacently run (on the same day) samples [for example, one sample yielding a high quantity of *16S* DNA (~400,000 copies in 750ng) was followed by a sample yielding a low quantity of *16S* DNA (50,000 copies in 750ng)]. This provides evidence that the samples were not contaminating each other during processing and reflects the thorough cleaning and decontamination of the ball mill instrument in between preparation of each liver powder. Second, we confirmed the presence of mycobacteria in some of the liver samples by extracting DNA from the samples with a different method: Bead beating the tissue in guanidine thiocyanate and phenol (**Supplementary Figure 2**). Notably, direct comparison of the ball mill vs bead beater on macaque liver tissue revealed that the DNA obtained from ball milling contained a higher *Mycobacterium* copy number than the same quantity of DNA obtained from bead beating, reflecting the higher efficiency of the ball mill in lysing this organism (**Supplementary Figure 2**).

The finding of high levels of atypical mycobacteria in the livers of the SIV+ and SIV+cART macaques was unexpected based on previous microbiome evaluations in macaques (32, 36–38). The *Mycobacterium* genus comprises hundreds of species that range from pathogens with significant clinical importance, such as members of the *M. tuberculosis* complex to NTM that are prevalent in water and soil (47). NTM, such as *M. smegmatis* are ubiquitous and inhabit a range of environmental reservoirs, including natural and municipal water, soil, aerosols, food and dust, with water being the most common source of infection (48). Overall, water treatment processes have been shown to efficiently remove mycobacteria, indicating that NTM recovered from water systems most likely contaminate post-treatment (49). Generally, environmental mycobacteria do not pose a risk to healthy individuals, but these NTM can cause disease in immunocompromised individuals. In one case study, an immunocompromised patient with an inherited interferon-gamma receptor deficiency was diagnosed with a mycobacterial infection identified as *M. smegmatis*, which proved fatal despite treatment (50). Interestingly, *M. smegmatis* has been shown to be pathogenic in other laboratory models; goldfish *M. smegmatis* infection induced recruitment of the bacterium to the liver and increased mortality (51). Remarkably, many opportunistic infections caused by *Mycobacterium* have been identified in HIV+ patients, particularly with members of the *M. avium* complex (MAC) (52). Early studies investigating the connection between HIV and NTM found that in patients with HIV there was a higher chance of isolating *M. xenopi* and *M. kansasii* from cultured respiratory secretions, in addition to *M. fortuitum*, *M. terrae*, and *M. scrofulaceum* from extrapulmonary sites (53). In fact, *M. kansasii* has been shown to cause serious pulmonary infections in patients with late-stage AIDS (54, 55). Despite the finding of opportunistic disease caused by NTM in HIV+ patients, previous studies have not identified significant levels of DNA from environmental mycobacteria in the gut microbiome of HIV+ patients, nor have previous SIV studies identified mycobacteria through *16S rRNA* gene sequencing in SIV+ macaques (56, 57). However, Sivanandham, et al. reported the presence of granulomas induced by atypical mycobacteria in the livers of SIV+ pig-tailed macaques (58), and He, et al. similarly reported the presence of granulomas in the livers of SIV+ African Green monkeys treated with a high fat diet although mycobacteria could not be identified by acid-fast staining (59). It is important to note that detection of *Mycobacterium* DNA requires specialized lysis steps to rupture the cell wall (60, 61). Utilization of the ball mill to mechanically disrupt liver tissue prior to DNA extraction in the present work likely enhanced the recovery of *Mycobacterium* DNA thereby explaining the prevalence of this taxon in our findings.

In previous work, we evaluated changes in the liver macrophage populations that expand during SIV infection and correlate with both pro-inflammatory (TNF-α, CCL3) and pro-fibrotic (TGF-β) mediators (41). Evaluating the CCL2-CCR2 chemokine network as an integral inducer of monocyte/macrophage infiltration into the liver, an upregulation of both CCL2 and CCR2 in the liver during untreated SIV infection was observed. This CCR2 expression positively correlated with the frequency of CD68+ macrophages, leading us to speculate that viral stimulation in the liver alters the immune environment through induction of CCL2, and possibly other chemokines, resulting in immune cell infiltration (41). However, in that study, we found that macrophage numbers do not correlate with bacterial load in the liver during SIV infection (41). That finding together with the increase in transcripts for neutrophil chemotactic mediators in *M. smegmatis*-stimulated hepatocytes herein, point to the possibility that neutrophils, another key phagocytic cell population, are involved in responding to bacteria in the SIV+ and SIV+cART liver. Neutrophils are rapidly recruited to sites of acute inflammation, though the method of recruitment of these cells to the liver is not well known (21, 22). Activated neutrophils can also promote disease progression *via* the secretion of pro-inflammatory cytokines (22). In HIV+ and HIV+cART patients, an increase in neutrophil frequency and survival was reported and correlated inversely with the ratio of *Lactobacillus* to *Prevotella* in the gut; *Lactobacillus* was associated with a decrease in neutrophil survival (62). Therefore, we hypothesize that the increased presence of NTM in the macaques may be correlated with increased levels of neutrophils.

Liver disease is currently a major contributor to morbidity and mortality in HIV+ and HIV+cART patients. Here, we identified microbial dysbiosis within the livers of SIV+ rhesus macaques, including heightened levels of atypical mycobacteria identified as *M. smegmatis*, or a close relative. Our data raise questions regarding a potential role for *Mycobacterium* in HIV+ people, including those on cART. Obtaining critical specimens, such as stool, liver and other tissues from these patients followed by optimized DNA extraction techniques is critical for determining the extent to which dysbiotic bacteria, including environmental mycobacteria, are part of the microbiome during HIV infection. Additional work in this area may aid in the development of chemoprophylaxis targeted to NTMs for HIV+ patients exhibiting liver disease.

## Data Availability Statement

The datasets presented in this study can be found in online repositories. The names of the repository/repositories and accession number(s) can be found below: https://www.ncbi.nlm.nih.gov/, PRJNA692039.

## Ethics Statement

The animal study was reviewed and approved by CIDR (SCRI) IACUC and WaNPRC IACUC.

## Author Contributions

BF and DS designed the study. BF, KF, AG, CF, and MW carried out the experiments. BF, BB, KF, AG, CF, MW, NK, and ND analyzed the data. MG and AG conducted the microbiome sequencing. BF and KF conducted the Nanostring analysis. JS coordinated and oversaw the animal work. BF, KF, ND, and DS wrote the paper. All authors contributed to the article and approved the submitted version.

## Funding

This project was supported by funds from the National Institute of Allergy and Infectious Diseases, National Institutes of Health grants R21AI100782 (DS) and R01AI134630 (DS). Research was also supported in part with funds from the National Institute of Allergy and Infectious Diseases, National Institutes of Health, including 5K22AI098440 (NK) and Contract No. HHSN272201300010C (MG), by the National Institutes of Health, Office of the Director P51OD010425 (MG) and under award P51OD010425 (Washington National Primate Research Center).

## Conflict of Interest

The authors declare that the research was conducted in the absence of any commercial or financial relationships that could be construed as a potential conflict of interest.

## Publisher’s Note

All claims expressed in this article are solely those of the authors and do not necessarily represent those of their affiliated organizations, or those of the publisher, the editors and the reviewers. Any product that may be evaluated in this article, or claim that may be made by its manufacturer, is not guaranteed or endorsed by the publisher.

## Supplementary Material

Supplementary Figure 1 Prevalent Genera Within the Macaque Liver. All prevalent genera identified within the 16s rRNA sequencing of all macaques in the study were plotted as a function of their overall prevalence within the dataset and with respect to their phylum. Genera plotted in order from left to right are 1. Mycobacterium, 2. Stenotrophomonas, 3. Delftia, 4. Massilia, 5. Acinetobacter, 6. Garnerella, 7. Halomonas, 8. Achromobacter, 9. Lactobacillus, 10. Staphylococcus, 11. Streptococcus, 12. Cloacibacterium, 13. Helicobacter, 14. Ottowia, 15. Phenylobacterium, 16. Corynebacterium_1, 17. Janibacter, 18. Tepidimonas, 19. Sphingomonas, 20. Micrococcus, 21. Prevotella_9, 22. Brevundimonas, 23. Holdemanella, 24. Hydrotalea, 25. Novosphingobium, 26. Psychrobacter, 27. Atopobium, 28. Bradyrhizobium, 29. Comamonas, 30. Haemophilus, 31. Nesterenkonia, 32. Novispirillum, 33. Pelomonas, 34. Actinomyces, 35. Dermacoccus, 36. Entrydobacter, 37. Lawsonella, 38. Mesthylobacterium, 39. Ralstoria, 40. Ruminococcaceae_UCG-005, 41. Stulliworthia, 42. Thermus, 43. Anaerococcus, 44. Chlamydia, 45. Cuthacterium, 46. Mycoplasma, 47. Pseudomonas, 48. Sneathia, 49. Prevotella_7.

Supplementary Figure 2 Comparison of DNA Extraction Methodologies on Liver Tissue. Frozen macaque liver samples were subjected to tissue dissociation and DNA extraction using either the Ball Mill (blue circles) or the Bead Beater (red squares). For equivalent input of the resulting DNA into 16s rRNA qPCR, resulting copies of the Mycobacterium 16s rRNA gene are shown, indicating the superior recovery of mycobacterial DNA by the Ball Mill tissue dissociation approach.
