# Supplementary figures and images for: Liver Bacterial Dysbiosis With Non-Tuberculosis Mycobacteria Occurs in SIV-Infected Macaques and Persists During Antiretroviral Therapy
Source: Front Immunol. 2022 Jan 10;12:793842. doi: 10.3389/fimmu.2021.793842 (PMC8784802; doi:10.3389/fimmu.2021.793842)

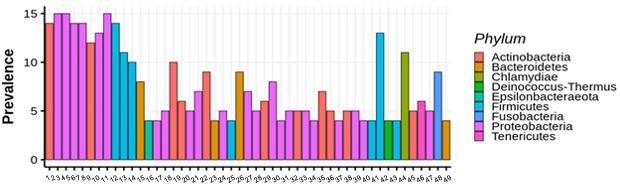

Supplement: Supplementary Figure 1 — Prevalent Genera Within the Macaque Liver. All prevalent genera identified within the 16s rRNA sequencing of all macaques in the study were plotted as a function of their overall prevalence within the dataset and with respect to their phylum. Genera plotted in order from left to right are 1. Mycobacterium, 2. Stenotrophomonas, 3. Delftia, 4. Massilia, 5. Acinetobacter, 6. Garnerella, 7. Halomonas, 8. Achromobacter, 9. Lactobacillus, 10. Staphylococcus, 11. Streptococcus, 12. Cloacibacterium, 13. Helicobacter, 14. Ottowia, 15. Phenylobacterium, 16. Corynebacterium_1, 17. Janibacter, 18. Tepidimonas, 19. Sphingomonas, 20. Micrococcus, 21. Prevotella_9, 22. Brevundimonas, 23. Holdemanella, 24. Hydrotalea, 25. Novosphingobium, 26. Psychrobacter, 27. Atopobium, 28. Bradyrhizobium, 29. Comamonas, 30. Haemophilus, 31. Nesterenkonia, 32. Novispirillum, 33. Pelomonas, 34. Actinomyces, 35. Dermacoccus, 36. Entrydobacter, 37. Lawsonella, 38. Mesthylobacterium, 39. Ralstoria, 40. Ruminococcaceae_UCG-005, 41. Stulliworthia, 42. Thermus, 43. Anaerococcus, 44. Chlamydia, 45. Cuthacterium, 46. Mycoplasma, 47. Pseudomonas, 48. Sneathia, 49. Prevotella_7. [file Image_1.jpeg]

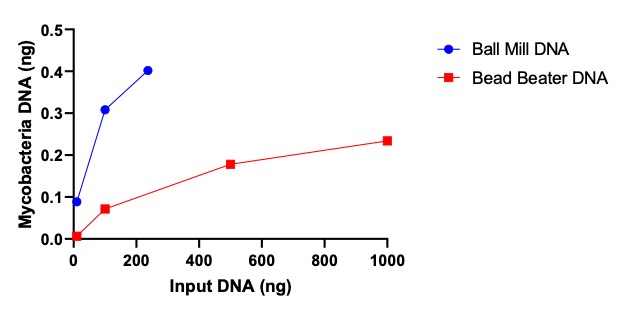

Supplement: Supplementary Figure 2 — Comparison of DNA Extraction Methodologies on Liver Tissue. Frozen macaque liver samples were subjected to tissue dissociation and DNA extraction using either the Ball Mill (blue circles) or the Bead Beater (red squares). For equivalent input of the resulting DNA into 16s rRNA qPCR, resulting copies of the Mycobacterium 16s rRNA gene are shown, indicating the superior recovery of mycobacterial DNA by the Ball Mill tissue dissociation approach. [file Image_2.jpeg]
