# Supplementary material for: Liver Bacterial Dysbiosis With Non-Tuberculosis Mycobacteria Occurs in SIV-Infected Macaques and Persists During Antiretroviral Therapy
Source: Front Immunol. 2022 Jan 10;12:793842. doi: 10.3389/fimmu.2021.793842 (PMC8784802; doi:10.3389/fimmu.2021.793842)
Supplement: Supplementary file 3 [file Table_1.docx]

**Supplementary Table 1. Primers and PCR conditions for species level identification of Mycobacteria.**

| **Gene** | **PCR Round** | **Primer Name** | **Primer Sequence** | **PCR Conditions** | **Size** |
| --- | --- | --- | --- | --- | --- |
| **16s** | 1 | Mycob For | 5’-ATG CAA GTC GAA CGG AAA GG-3’ | 1x - 94°C (5 min)  40x - 94°C (30 sec), 55°C (1 min), 72°C (1 min)  1x - 72°C (1 min) | 994bp |
|  |  | Mycob Rev | 5’-ACG TGT GTC CGG TGT TCC CT-3’ |  |  |
|  | 2 | MycobNest For | 5’-GAG TGG CGA ACG GGT GAG T-3’ | 1x - 94°C (5 min)  40x - 94°C (30 sec), 52°C (1 min), 72°C (1 min)  1x - 72°C (1 min) | 912bp |
|  |  | MycobNest Rev | 5’-CGT ACA GTT TGG GTC CAT TCC A-3’ |  |  |
| **rpoB** | 1 | rpoB For | 5’-CGA CCA CTT CGG CAA CCG-3’ | 1x - 94°C (5 min)  40x - 94°C (30 sec), 57°C (1 min), 72°C (1 min)  1x - 72°C (1 min) | 351bp |
|  |  | rpoB Rev | 5’-AGC TAG CCC GTG TAG GCC-3’ |  |  |
|  | 2 | rpoB  Nest For | 5’-CAC TTC GGC AAC CGC CGC C-3’ | 1x - 94°C (5 min)  40x - 94°C (30 sec), 59°C (1 min), 72°C (1 min)  1x - 72°C (1 min) | 339bp |
|  |  | rpoB  Nest Rev | 5’-CGT GAG GCC GGC ATC AC-3’ |  |  |
